# Gossypiboma Posing as a Diagnostic Dilemma: A Case Report and Review of the Literature

**DOI:** 10.1155/2014/713428

**Published:** 2014-12-15

**Authors:** K. N. Srivastava, Amit Agarwal

**Affiliations:** Department of General and Minimal Access Surgery, B. L. Kapur Super Speciality Hospital, Pusa Road, New Delhi 110005, India

## Abstract

The term gossypiboma is used to describe a retained surgical sponge after operation. It is a rare but serious complication which is seldom reported because of the medicolegal implications. Gossypiboma usually has varied and vague presentation and is also difficult to detect on radiological investigations. It can even remain silent and present years after the operation. We report a case of a 38-year-old lady who presented with vague pain and chronic lump in the right iliac fossa region. She had a history of cesarean section 4 years ago. Radiological investigations were inconclusive in detecting the retained sponge. A working diagnosis of mesenteric cyst was made and an exploratory laparotomy was done where she was found to have a large gossypiboma densely adhered to the small bowel and surrounding structures. Though rare, gossypiboma should be kept in mind as a differential diagnosis in postoperative cases presenting as vague pain or chronic lump even years after the operation.

## 1. Introduction

The term gossypiboma is used to describe a surgical sponge or a laparotomy pad left involuntarily in the body after a surgical procedure. The term is derived from a combination of Latin words “*Gossypium*” (cotton) and Swahili word “boma” (place of concealment) [1]. It is a rare surgical complication but can cause significant morbidity and mortality. Most gossypiboma cases are discovered during the first few days after surgery; however, they may remain undetected for many years [2]. Imaging modalities including plain radiography, ultrasonography (USG), computed tomography (CT), and magnetic resonance imaging (MRI) may help to have exact diagnosis [3]. Surgery is the recommended treatment option in these cases. Gossypiboma that presents late may pose a serious diagnostic dilemma. Gossypiboma should be considered as a diagnosis in patients with intra-abdominal mass with previous history of surgery.

## 2. Case Presentation

**Figure 1:**
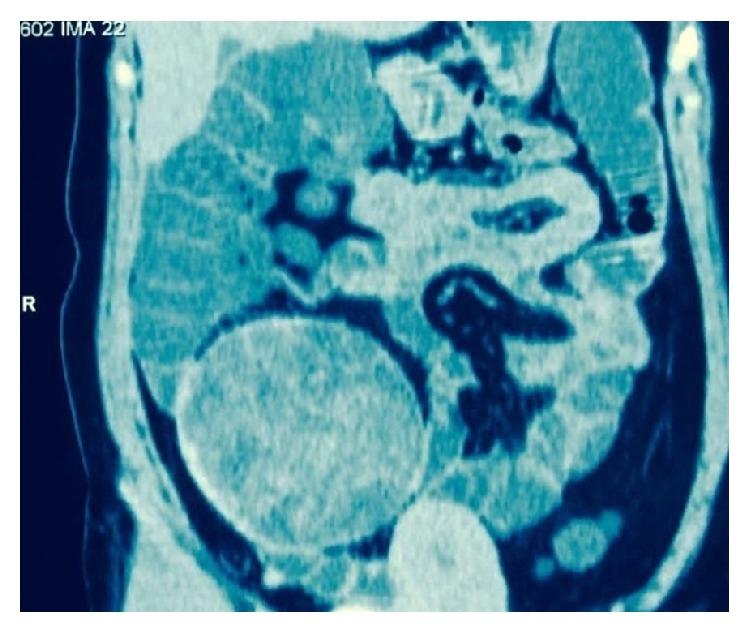
CECT image revealing the cyst in relation to the bowel.

A 38-year-old lady presented to our surgical clinics with the complaints of pain and lump right side lower abdomen 3 years ago. She had a history of emergency caesarean section done in a different country 4 years back. The patient had some discomfort at the right iliac fossa region in the postoperative phase which was explained to the patient as postoperative pain. No further advanced investigations were carried out at that stage. Following this the patient started to notice a lump formation at the site of the discomfort in the right iliac fossa region. The lump was gradually progressive in nature and the pain associated with the lump also gradually increased in intensity. The patient underwent an ultrasound and a CECT abdomen (Figure 1) before consulting us. Both these tests revealed a well-defined lump in relation to the small bowel in the right iliac fossa region suggestive of mesenteric cyst. On clinical examination there was a large lump in the right iliac fossa region of 20 cm × 15 cm. An MRI scan of the abdomen was conducted to ascertain the exact nature of the mass. The MRI was also inconclusive in making a definitive diagnosis and it revealed a cystic mass in relation to the bowel with a thick membrane inside the mass. A differential diagnosis of hydatid cyst was suggested upon the MRI.

**Figure 2:**
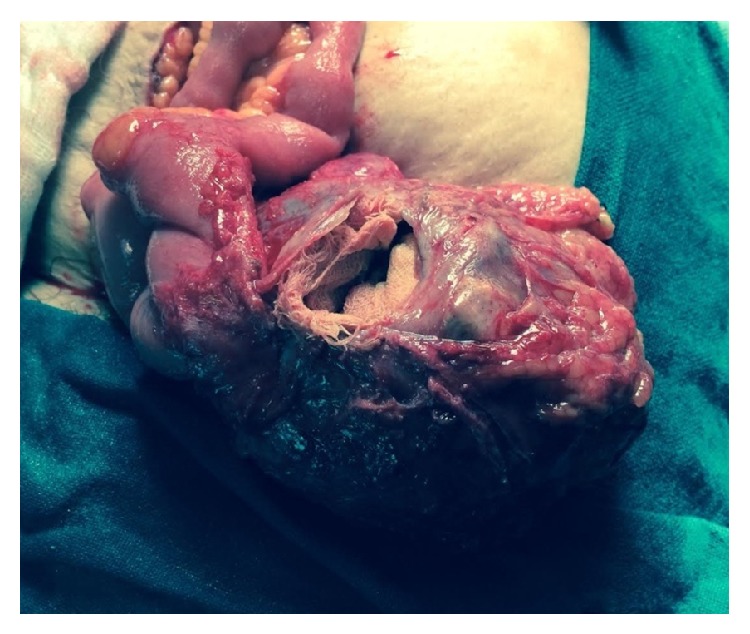
Intraoperative picture showing the lump adherent to the bowel with retained sponge seen inside.

**Figure 3:**
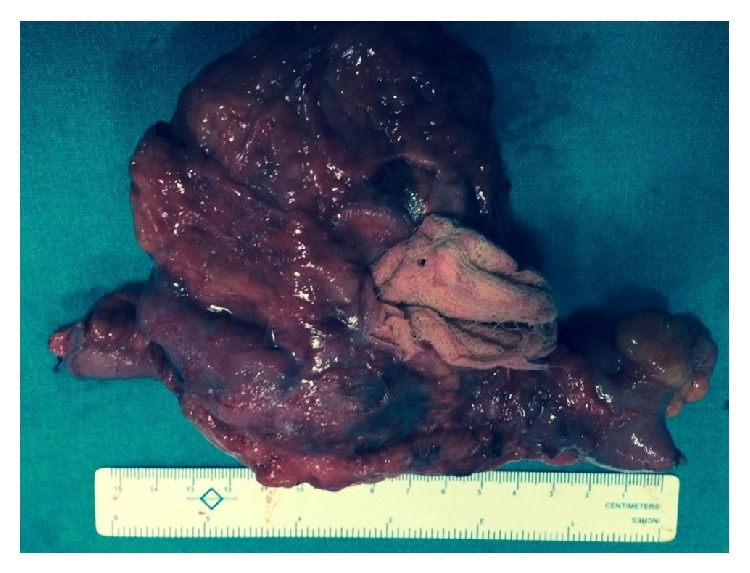
Picture of the resected specimen showing the retained sponge.

The patient was optimized and taken up for elective exploratory laparotomy. Upon laparotomy it was discovered that the lump in question was actually a tense encapsulated cystic mass of 20 cm × 15 cm × 14 cm densely adhered to the surrounding small bowel and omentum. The lump got open during dissection revealing thick pus along with a retained sponge as its contents (Figure 2). Resection of the mass along with a foot of the terminal ileum (Figure 3) was done. The postoperative period was uneventful and the patient was discharged on the seventh postoperative day.

## 3. Discussion

Gossypiboma or retained sponge is an important topic of discussion as it leads to significant embarrassment and can lead to humiliation and lawsuit as well. The reported incidence is between 1 in 1000–1500 abdominal operations [4]. However, the actual number is difficult to ascertain because of low reporting rate due to medicolegal implications [5].

Gossypiboma is most commonly seen in cases of emergency surgery, unexpected change in the surgical procedure, disorganization (e.g., poor communication), change in surgical team or scrub nurses, hurried sponge counts, long operations, unstable patient, inexperienced staff, inadequate staff numbers, and obesity [6].

The retained surgical sponge triggers two biological responses: aseptic fibrinous response due to foreign body granuloma or exudative reaction leading to abscess formation [7]. The symptoms depend upon the location, size of swab, and the type of reaction that occurs. Gossypiboma may present early with pain with or without lump formation or may remain asymptomatic for a long time with only vague symptoms. Patients may present with abdominal mass or subacute intestinal obstruction. Patients may rarely also present with fistula, perforation, or even extrusion per anus. In our case the gossypiboma caused vague symptoms for quite some time before ultimately resulting in a chronic lump formation.

Although radiological investigations are quite sensitive in picking up gossypiboma, they are limited in scope if the sponge does not have any radiological marker on itself. This is because the cotton can simulate hematoma, granulomatous process, abscess formation, cystic masses, or neoplasm [6]. Likewise in the case described here, the radiological investigations were not able to formulate the diagnosis as they were not able to pick up any radioopaque marker. This coupled with the chronic and nonspecific presentation poses a serious diagnostic dilemma. One has to have a high index of suspicion to be able to pick up this postoperative complication.

Prevention of gossypiboma is very important and can be done by simply keeping a thorough pack count during the course of the operation. Surgeons should perform a brief but thorough routine postoperative wound and cavity exploration prior to wound closure. It is now strictly recommended that only sponges with radioopaque makers be used. Newer technologies are being developed to decrease the incidence of gossypiboma, like radiofrequency chip identification by barcode scanner [8].

Treatment of gossypiboma is the surgical removal usually through the previous operative site, but endoscopic or laparoscopic approaches may be attempted. Due to chronicity of this disease and intense foreign body reaction, dense adhesion usually forms around the gossypiboma. As seen in our case the gossypiboma had formed a chronic lump which was so densely adhered to the small bowel that resection and anastomosis had to be done to remove the retained sponge.

## 4. Conclusion

Gossypiboma is a rare, avoidable, but serious, postoperative complication. It is usually asymptomatic and generally has nonspecific radiological findings. Hence the diagnosis is often delayed. Gossypiboma can cause wide variety of complications like perforation and adhesion to the adjacent structures. It can also be a cause for serious medicolegal problems.

It is best to avoid gossypiboma. The surgeons should comply with the current recommendations on the prevention of retained foreign bodies including use of radiological markers and routine pre- and postoperative sponge count.

Gossypiboma should be included in the differential diagnosis of soft-tissue masses or localized abdominal pains in patients with a history of a prior operation.
